# Use of fungal and bacterial protease preparations to enhance extraction of lipid from fish roe: Effect on lipidomic profile of extracted oil

**DOI:** 10.1016/j.fochx.2022.100499

**Published:** 2022-11-08

**Authors:** Mirja Kaizer Ahmmed, Alan Carne, Hong (Sabrina) Tian, Alaa El-Din Ahmed Bekhit

**Affiliations:** a Department of Food Sciences, University of Otago, P.O. Box 56, Dunedin 9054, New Zealand; b Department of Fishing and Post-harvest Technology, Faculty of Fisheries, Chittagong Veterinary and Animal Sciences University, Khulshi, Chittagong 4225, Bangladesh; c Department of Biochemistry, University of Otago, P.O. Box 56, Dunedin 9054, New Zealand; d Sanford Limited, 22 Jellicoe Street, Auckland 1010, New Zealand

**Keywords:** Alcalase, FP-II, HT, Proteases, Phospholipid, ^31^P NMR, Degree of hydrolysis, Omega-3 fatty acids

## Abstract

The present study investigated the hydrolysis of protein in hoki roe homogenate using a HT (bacterial), a FP-II (fungal) protease preparations and Alcalase (bacterial) to enhance lipid yield extraction. The degree of hydrolysis was determined at various pH, temperature and time using casein and hoki roe. Total lipid extraction and lipidomic analysis was carried out following proteolysis of hoki roe homogenate. The degree of hydrolysis and SDS-PAGE revealed that the hydrolytic capability of Alcalase was better than HT and FPII. The total extracted lipid yield was better following hydrolysis with Alcalase (19.29 %), compared to HT (18.29 %) and FPII (18.33 %). However, the total phospholipid (PL) and n-3 fatty acid yields were better from HT hydrolysed hoki roe homogenate (PL = 30.7 μmol/g; n-3 = 10.5 %), compared to Alcalase (PL = 22 μmol/g; n-3 = 5.95 %). Overall, this study indicates that HT protease preparation hydrolysis of fish roe homogenate can both enhance lipid extraction and retain lipid quality.

## Highlights

• Lipid extraction of fish roe was evaluated after hydrolysis with HT, FP-II and Alcalase proteases.
• Alcalase hydrolysis of fish roe protein was more extensive than that of HT and FP-II.
• The highest total lipid yield was obtained following hydrolysis of fish roe with Alcalase.
• Alcalase hydrolysis achieved the greatest degree of hydrolysis and yielded less oxidised lipid.
• The yield of omega-3 fatty acids and phospholipids was highest after HT hydrolysis.

## Introduction

Fish roe contains high levels of n-3 phospholipids, which is reported to have substantial health benefit for brain and general health (Ahmmed, Ahmmed, Tian, Carne, & Bekhit, 2020). Hoki roe contains a considerable amount of phospholipid and long chain n-3 fatty acids (Ahmmed, Bunga et al., 2020). Therefore, hoki roe was selected for this study to investigate the ability of enzyme-assisted extraction to enhance the lipid extraction yield from hoki roe. Several protease preparations including Neutrase, Alcalase and Protamex have been used for proteolysis prior to lipid extraction from fish and fish co-products (Dumay et al., 2004, Gbogouri et al., 2006, Šližytė et al., 2005). However, the extraction efficiency of different protease preparations can vary. Gbogouri et al. (2006) compared the efficiency of pre-treatment with different proteolytic food-grade protease preparations (Alcalase, Neutrase and Protamex) in the extraction of lipids from salmon head, and obtained the highest lipid recovery using Alcalase (19.6 %) compared to Neutrase (14.4 %) and Protamex (14.6 %). Enzymatic hydrolysis using papain, chymotrypsin, Protamex and Flavourzyme was reported to increase the yield of total extracted lipid and phospholipid of cod liver and roe (Dumay, Barthomeuf, & Bergé, 2004). Alcalase was reported to be better than Lecitase Ultra for proteolysis of raw cod co-products (Šližytė, Rustad, & Storrø, 2005), and was found to generate hydrolysates with high antioxidant activity from cod frame (Bordbar, Ebrahimpour, Zarei, Abdul Hamid, & Saari, 2018). Alcalase has also been used to achieve enhanced extraction of lipids from cuttlefish co-products (Kechaou et al., 2009) and trout roe (Mahmoud et al., 2008). In the present study, Alcalase was selected as a positive control reference for comparison with commercial bacterial protease HT and a fungal protease FP-II preparations.

The HT and FP-II food-grade protease preparations have been reported to be effective in hydrolysing meat myofibrillar and connective tissue proteins (Ryder, Ha, Bekhit, & Carne, 2015), and for generation of bioactive peptides from meat industry co-products (Ryder, Bekhit, McConnell, & Carne, 2016). However, these particular protease preparations have not been used for fish roe protein hydrolysis, to evaluate the effect of hydrolysis on the extracted lipidomic profile of fish roe.

Therefore, the present study aimed to investigate (i) the hydrolytic capability of the HT and FP-II protease preparations compared to that of Alcalase, on proteolysis of hoki roe homogenate, and (ii) compare the lipidomic profile (lipid yield, quality, fatty acid composition and phospholipid composition) of lipid extracted from the proteolyzed hoki roe homogenate. The overall research hypothesis was that different protease preparations will exhibit different hydrolytic capability toward the protein in hoki roe homogenate, that could potentially enhance the extraction of lipid in terms of lipid yield, fatty acid and phospholipid composition and lipid quality.

## Materials and methods

### Chemicals

All chemicals used were of analytical reagent grade as reported previously (Ahmmed, Bunga et al., 2020). Alcalase protease solution was obtained from Sigma-Aldrich, Auckland, New Zealand. FP-II and HT protease preparation powders were supplied by McConnell Bros. (formerly Enzyme Solutions Pty. ltd.) (Croydon South, Victoria, Australia). These proteases are referred to as ‘protease preparations’ as they are not single pure proteases, (Ha et al., 2012, Ryder et al., 2015, Ryder et al., 2016). Casein standard (Sigma-Aldrich, Auckland, New Zealand) and hoki roe (obtained from Sanford, New Zealand) were used as substrates. Casein was prepared in 50 mM potassium phosphate buffer (pH 7.5) to generate a stock solution of 6.5 mg/mL. The casein solution was sub-aliquoted into 15 mL Falcon tubes and stored at −20 °C for further use. A total of 30 hoki roe were cut into smaller pieces and randomized into three pooled batches, which were considered as individual biological replicates. Each batch of roe was homogenized on ice separately using a bench-top lab blender (Watson Victor Limited, Wellington, New Zealand). After each homogenization process, the blender was washed with pressurized water and cleaned. The homogenised fish roe samples were stored at −20 °C until further use.

### Characterization of the protease preparations

Both casein, and the hoki roe homogenate were used as substrates to investigate the hydrolytic capability of Alcalase, HT and FP-II.

#### Total protein measurement

The total protein content of each commercial protease preparation, and substrates (casein and hoki roe homogenate), were determined using the bicinchoninic acid (BCA) total protein assay, using a microplate reader according to the manufacturer’s instructions. The reagents used in the BCA assay include Pierce™ BCA reagent A (containing (w/v) 1 % BCA, 2 % Na_2_CO_3_·H_2_O, 0.16 % sodium tartrate, 0.4 % NaOH, 0.95 % NaHCO_3_), and Pierce™ BCA reagent B containing 4 % (w/v) CuSO_4_·5H_2_O (Thermo Fisher, Waltham, MA, USA), and Triton™ X-100 (Merck KGaA, Darmstadt, Germany). BCA assay reagent was prepared just before use by mixing 48 parts BCA Reagent A with 1 part BCA Reagent B and 1 part Triton X-100. A 20 µL sample (casein, or hoki roe homogenate, or protease, or BSA protein standard), was transferred into a transparent 96 well flat bottom microplate. Freshly prepared BCA reagent (200 μL) was added into each well containing samples. Aluminium foil was used to cover the plate prior to incubation at 37 °C for 30 min. The microplate was cooled to room temperature for 5 min, and then read at 562 nm using a microplate reader. Bovine serum albumin (BSA) (Merck KGaA, Darmstadt, Germany) was used to prepare a standard curve of 0–20 μg).

#### Determination of the protease preparations activities using casein as a substrate

The casein hydrolysing activity of the protease preparations was determined at 37 °C) and pH 7.5, according to a standard method recommended by Sigma chemical company as described by Cupp-Enyard (2008). Hoki roe homogenate was used as a substrate to investigate the hydrolytic activity of the three commercial protease preparations (Alcalase, HT and FP-II), to determine the amount of each protease that produces a comparable degree of hydrolysis, which was applied to subsequent studies.

The HT and FP-II protease preparation powders were prepared as 2 mg/mL stock solutions in 10 mM sodium acetate buffer, 5 mM calcium, pH 7.5 (Cupp-Enyard, 2008). The stock solutions were subsequently diluted to 0.02 mg/mL for use using the same sodium acetate buffer. Alcalase was diluted with sodium acetate buffer to prepare a stock of 10 µL/mL, which was subsequently diluted to 0.01 µL/mL for use.

For the caseinolytic assay, aliquots of casein solution were transferred into 1.7 mL microcentrifuge tubes. Each tube was incubated at 37 °C for 10 min to equilibrate, and then 100 µL of either Alcalase (0.01 µL/mL), FP-II (0.02 mg/mL) or HT (0.02 mg/mL) stock solutions were added to separate casein containing microcentrifuge tubes and vortexed for 30 s before being placed in a shaker incubator. A control set that contained no protease was assayed in parallel. After 10 min of incubation at 37 °C, 500 µL of 110 mM trichloroacetic acid (TCA) was added to stop the reaction. With the control samples, after incubation and addition of TCA, 100 µL of protease stock was added individually to corresponding controls. The samples were centrifuged at 21,000*×g* for 20 min and a 200 µL aliquot of the supernatant was transferred into a clean 1.7 mL microcentrifuge tube and 500 µL of Na_2_CO_3_ (500 mM) was added, followed by addition of 100 µL Folin-Ciocalteu’s phenol reagent (0.5 M) to each tube. The tubes were incubated at 37 °C for 30 min using a shaker incubator. After incubation the tubes were centrifuged at 21,000*×g* for 10 min. A 200 µL aliquot of the supernatant was transferred into a well of a 96 well transparent flat-bottom microplate and the sample absorbance was read at 660 nm using a microplate reader. Different dilutions (0–0.44 μmol) of a 1.1 mM tyrosine stock solution were used to construct a standard curve.

The following equation was used to calculate the caseinolytic proteolytic activity in terms of tyrosine equivalent units (Cupp-Enyard, 2008)

$$Units/mL\;\;protease=\frac{\mu mol\;\;tyrosine\;\;equivalents\;\times \;VT\;}{\mathrm{VE}\times \;T\times \;VA}$$

VT = Total volume (in mL) of assay.

VE = Volume of protease (mL).

T = Time of assay (min) as per the unit definition.

VA = Volume (ml) used in colorimetric determination.

Protease activity was also determined using hoki roe homogenate as a substrate following the overall same procedure as for casein. Hoki roe homogenate was diluted (roe:buffer = 1:3) using 50 mM potassium phosphate buffer, pH 7.5. The buffer diluted roe homogenate sample was further diluted to achieve a stock of 6.5 mg protein/mL, using the potassium phosphate buffer to make the protein concentration equivalent to that of the casein stock solution (6.5 mg/mL). The total protein concentration of the diluted samples was determined by the BCA method.

#### Investigation of the effect of pH on the proteolytic activity of the protease preparations

Protease activity was also determined at four different pH values (5.0, 6.0, 7.0, and 8.0) at a specific temperature (45 °C) to investigate the effect of pH on the protease activity. At 45 °C, the three protease preparations (Alcalase, FP-II and HT) are reported to be moderately active (Mahmoud et al., 2008, Ryder et al., 2016), and at this temperature lipid quality is maintained (Franklin, Haq, Roy, Park, & Chun, 2020). Both casein and hoki roe homogenate were assayed as substrates. The different pH of hoki roe homogenate aliquots was achieved by mixing hoki roe with pH 5, 6, 7 and 8 phosphate buffer in a ratio of 1:3). Alcalase, FP-II and HT stock solution was adjusted to the target pH prior to incubation. The proteolysis reactions were incubated for 10 min (Cupp-Enyard, 2008), and then the proteolysis was stopped by adding 0.5 mL of 20 % (w/v) TCA and the proteolytic activity was determined as described above.

#### Investigation of the effect of temperature on the proteolytic activity of protease preparations

Hydrolysis of the casein and hoki roe homogenate substrates was also analyzed at different temperatures (35, 45, 55 and 65 °C), at pH 7.0, based on information obtained from an effect of pH experiment. Aliquots (100 µL) of the protease stocks were added individually to separate microcentrifuge tubes containing either casein or hoki roe homogenate (500 µL). The protease and substrate mixture was vortexed for 30 s and incubated for 10 min at either 35, 45, 55 or 65 °C. The reaction was stopped by adding 500 µL TCA stock and proteolysis activity was determined as described above.

#### Determination of degree of hydrolysis

##### Using casein as a substrate

The casein substrate stock (6.5 mg/mL) was hydrolysed at a specific temperature of 45 °C and a pH of 7.0, using a specific amount of either Alcalase (0.01 µL/ mL), FP-II (0.02 mg/mL), or HT (0.02 mg/mL), and the reaction kinetics were followed over 4 h. The temperature (45 °C) and pH (7.0) were selected based on the results obtained from an analysis of pH and temperature. Time course aliquots (600 µL) were collected every 20 min from the hydrolysates and 500 µL of 110 mM TCA was added to inactivate the protease. The assay solution was centrifuged at 21,000*×g* for 20 min and an aliquot of the supernatant was transferred to a 1.7 mL microcentrifuge tube. The supernatant was centrifuged again for 10 min at 21,000*×g*, and then 10 µL of the supernatant was mixed with 990 µL water to dilute the sample, followed by either 25 µL of diluted assay supernatant, or l-serine standard (1 mg/mL) was transferred into a transparent flat-bottom microplate and 200 µL of OPA (*o*-phthaldialdehyde) reagent was added, to determine the degree of hydrolysis (Aspmo et al., 2005, Church et al., 1983). The OPA reagent was freshly prepared before each assay by mixing 50 mL of 0.1 M sodium tetraborate buffer, 5 mL of sodium dodecyl sulphate (20 %, w/v), 80 mg of OPA (pre-dissolved in 1 mL of methanol), and 200 µL of β-mercaptoethanol, the volume was made to 100 mL with milli-Q Type 1 water, and the solution was mixed. The assay was conducted in the dark as the reagent is light sensitive.

To investigate the effect of protease concentration on the degree of casein hydrolysis, different amounts of Alcalase (150U/mL, 1500U/mL, 15,000U/mL, or 70,000U/mL) were used in assays, and the reaction was followed over 3 h. Sampling of the reaction was carried out at 20 min intervals and the degree of hydrolysis determined using the OPA assay.

##### Using hoki roe as a substrate

Time course hydrolysis was also carried out using hoki roe homogenate as a substrate with the reaction followed for 24 h, and the degree of hydrolysis of time course samples determined by OPA assay (Aspmo et al., 2005, Church et al., 1983). The same incubation temperature (45 °C) was used based on the findings from the degree of hydrolysis assay. The amount of protease used was based on the results of the caseinolytic activity. The degree of hydrolysis was determined at three different amounts of protease (1500U, 15,000U, or 30,000U) (Table S1). A mixture of 10 mM sodium acetate, 5 mM calcium, pH 7.0, was used for protease stock solution preparation.

#### SDS-PAGE analysis of protein hydrolysis profile

One dimensional sodium dodecyl sulphate polyacrylamide gel electrophoresis (1D SDS-PAGE) was used to display the protein hydrolysis profiles of hydrolysed hoki roe homogenate protein, using Bolt gradient (4–12 %) Bis-Tris gels (Ryder et al., 2015). An aliquot (20 µL) of a protein containing sample was added to 7.6 µL Bolt LDS sample buffer containing lithium dodecyl sulphate (LDS) at pH 9.5 and 3.0 µL Bolt™ sample reducing agent containing 500 mM dithiothreitol (DTT). All Bolt reagents were purchased from Invitrogen Life Technologies, Thermo Scientific, Auckland, New Zealand. A programmable Thermal Cycler (Perkin-Elmer, USA) was used to incubate the samples for 5 min at 70 °C, then cooled before loading in gel lanes. Novex Sharp Pre-Stained protein standard (Invitrogen) was loaded in one lane. The electrophoresis was carried out at room temperature in 1 × Bolt MES SDS running buffer for 35 min at 165 V. After electrophoresis, the gel was washed with milli-Q Type 1 water four times, each for 10 min, and then stained overnight with gentle shaking in 20 mL SimplyBlue SafeStain (Invitrogen) followed by destaining in water, and an image was captured with a Canon CanoScan LiDE 600F scanner.

### Lipidomic profile of lipid obtained using protease-assisted extraction

#### Enzymatic hydrolysis of hoki roe homogenate under optimal condition

Three samples of hoki roe homogenate (20 g) (prepared as described above) were each mixed with 60 mL100 mM Tris-HCl buffer, pH 7.0, by vortexing for 30 s. Aliquots of the diluted stock containing 20 g hoki roe homogenate were hydrolysed with three different protease preparations (Alcalase, FP-II or HT) at three different concentrations (1, 2 and 4 % of the substrate), Alcalase (200, 400, or 800 µL); HT (200, 400, or 800 mg) and FP-II (200, 400, or 800 mg) for 3 h at a specific temperature (45 °C) and pH (7.0). The concentration range of the protease preparations used was selected based on previous studies (Mahmoud et al., 2008, Normah et al., 2005, Shu et al., 2016), where 1–4 % protease preparations (Alcalase, Neutrase and Protamex) were used for lipid extraction from trout roe (Mahmoud et al., 2008), hydrolysis of goat milk casein (Shu et al., 2016), and fish hydrolysate preparation (Normah et al., 2005), as well as consideration of results obtained in the present study. After enzymatic hydrolysis, the samples were frozen immediately by immersion in liquid nitrogen. The frozen samples were freeze-dried for 3 d to remove the moisture from the hydrolysed sample. The freeze-dried samples were used for lipid extraction.

#### Lipid extraction

The total lipid was extracted using a hexane/ethanol based solvent extraction method, ETHEX (Ahmmed, Bunga et al., 2020). The total extracted lipid was weighed and stored at −80 °C for further analysis.

#### Determination of lipid oxidation using thiobarbituric acid reactive substances (TBARS) and peroxide value (PV)

Lipid oxidation was determined using the thiobarbituric acid (TBA) reactive substances (TBARS) method as described by Xia, Kong, Liu, and Liu (2009). The detailed method of sample preparation and calculation of TBARS (mg malonaldehyde equivalent/kg of lipid) has been discussed in Ahmmed et al. (2022). The PV value was determined as described by Sakanaka et al. (2004). The detailed method of sample preparation and calculation of peroxide value (milliequivalent peroxide/kg lipid) were carried out as reported earlier (Ahmmed et al., 2022).

#### Fatty acid methyl ester (FAME) analysis

The total lipid extracted from freeze-dried hoki roe homogenate hydrolysates was used for the preparation of FAME. The FAME was analyzed by GC-FID. The detailed sample preparation and GC running conditions were those described previously (Ahmmed, Bunga et al., 2020).

#### Phospholipid composition

The total lipid was mixed with 1 mL of sodium cholate/D_2_O/EDTA based buffer (pH 7.4) containing 1 μmol/mL glyphosate as internal standard. Detailed sample preparation and NMR running conditions were carried out as described previously (Ahmmed, Bunga et al., 2020). The sample was transferred into a 5 mm NMR tube and ^31^P NMR analysis was carried out using a Varian MR400 NMR instrument (Agilent Technologies, California, USA).

### Statistical analysis

Statistical analysis was performed with Minitab® Software (Version 16.0, Minitab Inc., Pennsylvania, USA). The results from the protease assays were from three independent experiments in which the measurements were carried out in triplicate. The homogeneity of variance and normality of the data were determined using Bartlett’s test and the Shapiro-Wilk test, respectively. Analysis of variance (ANOVA) was carried out using General Linear Model (GLM) in Minitab® version 15.1.0 (Minitab Limited, Sydney, Australia) to determine the effects of protease, concentration, pH and temperature on the measured parameters. The means were separated using Tukey’s test at 0.05 level of significance.

## Results and discussion

### Total protein content of the protease preparations used for hydrolysis

The total protein content of the three protease preparations (Alcalase, FP-II and HT) used for hydrolysis of casein and hoki roe homogenate are shown in Table 1. The results show a higher protein content for the HT protease (512 ± 15.28 mg/g dry weight commercial powder) followed by FP-II (389 ± 36.12 mg/g dry weight commercial powder) and Alcalase (124.9 ± 24.1 mg/g). In the present study, the total protein percentage in FP-II commercial powder (40 %, w/w), was found to be slightly lower than that reported for a different batch of FP-II commercial powder (44 %, w/w) used by Ryder et al. (2015). As mentioned above, the commercially supplied HT and FP-II proteases are essentially protease containing extracts, not purified proteases, as they contain a number of proteins. In addition, there has been some variation in different batches of the commercial proteases that we have obtained.

**Table 1:** Specific activity (μmol/min/g substrate protein) of Alcalase, FP-II and HT protease preparations at pH (7.0) and temperature (45 °C), and total protein.

Protease preparations	Protease concentration*	Units/mL Protease solution	Total protein (mg/g)^1^	Specific activity μmol/min/g substrate protein
*Casein as substrate*				
Alcalase	0.01 µL/mL	1.89 ± 0.05	124.9 ± 24.1	12.6 × 10^5^ ± 0.47 × 10^5^
FP-II	0.02 mg/mL	2.13 ± 0.01	389 ± 36.12	4.1 × 10^5^ ± 0.15 × 10^5^
HT	0.02 mg/mL	2.43 ± 0.06	512 ± 15.28	4.88 × 10^5^ ± 0.08 × 10^5^

*Hoki roe homogenate as substrate*				
Alcalase	0.01 µL/mL	1.53 ± 0.01	124.9 ± 24.1	10.5 × 10^5^ ± 0.47 × 10^5^
FP-II	0.02 mg/mL	1.44 ± 0.02	389 ± 36.12	3.7 × 10^5^ ± 0.53 × 10^5^
HT	0.02 mg/mL	2.50 ± 0.06	512 ± 0.15	4.86 × 10^5^ ± 0.08 × 10^5^

* Alcalase is provided commercially as a solution. Concentration of the prepared stock solution of Alcalase (µL/mL), HT (mg/mL) and FP-II mg/mL) as mentioned in the second column.

1 Alcalase (mg/g equivalent Alcalase solution); HT and FP-II (mg/g dry powder).

### Hydrolytic activity of the protease preparations used for hydrolysis

The specific activity of the three protease preparations (Alcalase, FP-II and HT) used for hydrolysis of hoki roe homogenate is shown in Table 1. HT was found to have a higher specific activity than FP-II, both for casein and the hoki roe homogenate substrate (Table 1). This result is in agreement with the findings of Ryder et al. (2015), who reported a higher caseinolytic specific activity for HT protease (4.4 × 10^6^ ± 2.1 × 10^5^ fluorescence/min/mg protein) compared to that of FP-II (2.4 × 10^6^ ± 6.0 × 10^4^ fluorescence/min/mg protein). The Alcalase used in the present study was found to have a higher specific activity (12.6 × 10^5^ ± 0.47 × 10^5^ μmol/min/g substrate protein) compared to that of FP-II (4.1 × 10^5^ ± 0.15 × 10^5^ μmol/min/g substrate protein) and HT (4.88 × 10^5^ ± 0.08 × 10^5^ μmol/min/g substrate protein), for hydrolysis of both casein and hoki roe homogenate. Alcalase was reported to have a higher proteolytic activity than Neutrase and Protamex with rainbow trout roe as a substrate (Mahmoud et al., 2008). Also, Alcalase was found to be more active than Lecitase Ultra for hydrolysis of raw cod co-products (Šližytė et al., 2005). HT and FP-II are both reported to contain a mixture of endo- and *exo*-proteases (Ryder et al., 2015), whereas the main protease component of Alcalase is reported to be an *endo*-protease (Thiansilakul, Benjakul, & Shahidi, 2007). In the present study, Alcalase was found to contain one main protein component by SDS-PAGE. All three of the protease preparations used in the present study exhibited higher protease activity using casein as a substrate compared to hoki roe homogenate. Hoki roe is a complex natural matrix containing other chemical constituents, such as lipid, ash, and carbohydrate, in addition to the protein fraction and has a different molecular arrangement compared to a casein fraction isolated from milk, hence differences in hydrolysis of casein compared to hoki roe homogenate could be expected. Fish roe is composed of a particular structure, which is reported to inhibit fungal and microbial attack in aquatic systems (Mahmoud et al., 2008). Further, isopeptide bonds and disulphide bonds are reported to be present (Papadopoulou, Galanopoulos, & Hamodrakas, 1996), which could affect hydrolysis by protease preparations.

### Effect of pH and tempeature on protease activity

**Fig. 1:**
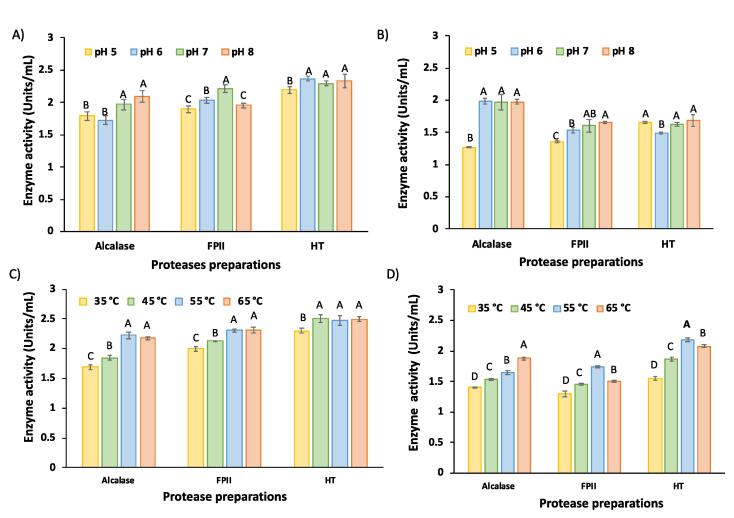
Effect of different pH (A, B) and temperature (C, D) on protease activity (Units/mL protease), with casein (A, C) and hoki roe homogenate (B, D) as substrates. The assay was incubated for 10 min and the enzyme activity was determined using the OPA method.

To obtain information about protease activity as a function of pH and temperature for the two substrates (casein and hoki roe homogenate), the proteolytic activity was further evaluated at four different pH (5, 6, 7, and 8) and temperatures (35, 45, 55, and 65 °C) as summarised in Fig. 1.

The pH and temperatures were chosen based on information provided by the commercial suppliers of the protease preparations, and taking into consideration the need to carry out the hydrolysis under as mild conditions as possible, as the overall aim was to achieve proteolysis of hoki roe homogenate, and minimise lipid modification. The results show that there is a significant effect (*p* < 0.05) for pH (Fig. 1A and 1B) and temperature (Fig. 1C and 1D) on the protease activity. All three protease preparations evaluated were found to be substantially active at pH 5–8 and at 55–65 °C, for both of the substrates, in agreement with Ryder et al. (2015). The activity of the protease preparations is lower with the hoki roe homogenate (Fig. 1B and 1D), compared to the casein protein (Fig. 1A and 1C) substrates.

A variety of conditions have been reported in the literature for hydrolysis of fish protein from various sources using Alcalase. Examples include hydrolysis of trout roe protein at pH 7.5 and 40 °C (Mahmoud et al., 2008), hydrolysis of yellowfin tuna protein at pH 8.0 and 50 °C (Guérard, Dufosse, de La Broise, & Binet, 2001), and hydrolysis of common carp roe at pH 8.0 and 55 °C (Chalamaiah et al., 2015). In the present study, a slightly lower temperature (45 °C) and neutral pH (7.0) were used for all protease preparations, as the main aim of this research was to achieve hydrolysis of protein to investigate whether an enhanced yield of lipid could be obtained, without compromising extracted lipid quality. Fish lipid contains a high amount of polyunsaturated fatty acid (PUFA) and phospholipid, which are more stable at neutral pH and lower temperature (Ahmmed, Ahmmed et al., 2020). Elevated temperature has been reported to cause considerable degradation of n-3 fatty acids, lipid oxidation and generation of free radicals (Ghnimi, Budilarto, & Kamal-Eldin, 2017). Ryder et al. (2015) and Ha et al. (2013) found protease activity was maximal for fungal protease preparations at 45 °C. The results in the present study indicate that all three of theprotease preparations are sufficiently active at pH 7.0 and 45 °C, to achieve hydrolysis of both the casein and hoki roe homogenate protein.

### Degree of hydrolysis

**Fig. 2:**
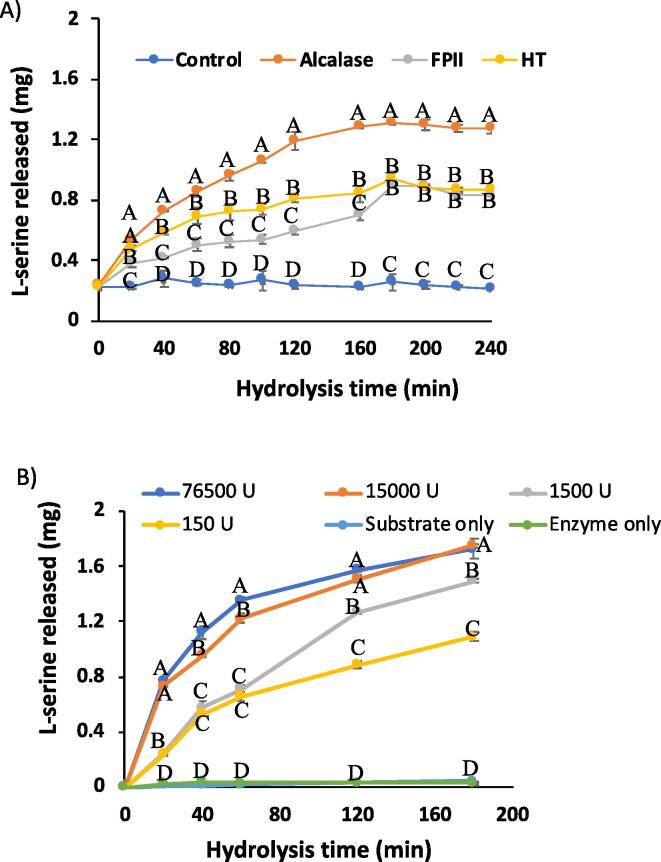
(A) Hydrolysis of casein by Alcalase, HT and FP-II protease preparations, (B) Hydrolysis of casein using different amounts of Alcalase protease preparation (150 U, 1500 U, 15,000 U, and 76,500 U), as determined by degree of hydrolysis. Different letters (A-D) on the line graph indicate significant differences (*p* < 0.05) in l-seine released (mg) among the different treatments at a specific time point.

The protease activity was determined based on a fixed time incubation for the evaluation of the effect of pH and temperature, which is presented in the previous section. As the degree of hydrolysis will vary with incubation time (Ryder et al., 2015), the degree of hydrolysis was determined by assaying time course (0, 20, 60, 80, 100, 120, 160, 180, 200, 220, and 240 min) hydrolyses of casein and hoki roe homogenate as substrates with Alcalase, HT and FP-II protease preparations conducted at pH 7.0 and 45 °C. Fig. 2 summarises the results of the time course hydrolyses conducted.

#### Casein as a substrate for protease hydrolysis

All of the protease preparations, at specified concentration (Alcalase, 0.01 μL/mL; HT, 0.02 mg/mL and FP-II protease, 0.02 mg/mL), achieved near maximal hydrolysis capability of casein substrate within 180 min (Fig. 2A), based on flattening of the hydrolysis time course curves. As the rate and extent (degree) of hydrolysis can vary depending on protease concentration (Shu et al., 2016), further time course hydrolyses (0, 20, 60, 80, 100, 120, 160, and 180 min) were carried out using different amounts of Alcalase (150 U, 1500 U, 15,000 U, and 70,000 U), with casein as substrate. The concentration effect was evaluated with Alcalase and casein, and then applied to all three protease preparations subsequently (Fig. S1), using roe homogenate, which was the main substrate of interest.

Casein-only and protease-only controls indicated that there was no generation of amino groups over the hydrolysis period of 180 min (Fig. 2B). The results show that a higher amount of protease resulted in more protein hydrolysis (Fig. 2).

#### Hoki roe as a substrate for protease hydrolysis

Following the experiments with casein hydrolysis, hoki roe homogenate protein was hydrolysed for 24 h at a specific temperature and pH with different amounts of each of the Alcalase, HT and FP-II protease preparations (1500 U/mL, 15,000 U/mL, and 30,000 U/mL), and the degree of hydrolysis over the incubation time course was determined by measuring release of TCA-soluble peptides using the OPA assay. Extended hydrolysis was carried out to investigate maximum hydrolysis with each protease. The results indicate that all of the protease preparations used in the present study were capable of hydrolysing the hoki roe homogenate protein under the stated experimental conditions, and substantial hydrolysis was achieved by 3 h, as further hydrolysis time leads to a plateauing of the degree of hydrolysis curve, indicating no substantial further increase in protein hydrolysis. It was also of interest to limit the time that the lipid containing roe homogenate was exposed to elevated temperature, to minimise the possibility of temperature related lipid modifications. It has been reported that a high proportion of susceptible peptide bonds are hydrolysed early in a protease hydrolysis (Normah et al., 2005), and that proteolytic hydrolysis for an extended period may also lead to product inhibition, where peptides compete with the remaining substrates for the binding sites of the protease (Luo, Chen, Boom, & Janssen, 2018). Mahmoud et al. (2008) reported achieving 7.8 % hydrolysis of rainbow trout roe protein within 120 min, using Alcalase, which was found to enhance both the total lipid and phospholipid extracted yield.

The results obtained also indicate that the degree of hydrolysis increased with the increase of protease concentration (Fig. S1A, S1B and S1C). Alcalase was found to have the highest hydrolysis capability followed by the HT and FP-II protease preparations (Fig. S1D). It has been reported that endopeptidases such as Alcalase are capable of hydrolysing the roe protein more than protease preparations containing a endo- and exo-peptidase enzyme mixture, such as Flavourzyme (Hrckova et al., 2002, Thiansilakul et al., 2007). Alcalase has been reported to exhibit a higher degree of hydrolysis capability of cuttle fish and sardine viscera compared to that of Protamex and Flavourzyme (Kechaou et al., 2009). The hydrolysis capability of the three protease preparations in the present study was investigated by analysing the protein hydrolysis profile of the time course hydrolysates using 1D-SDS-PAGE.

### 1D-SDS-PAGE protein profile of hoki roe homogenate hydrolysis

**Fig. 3:**
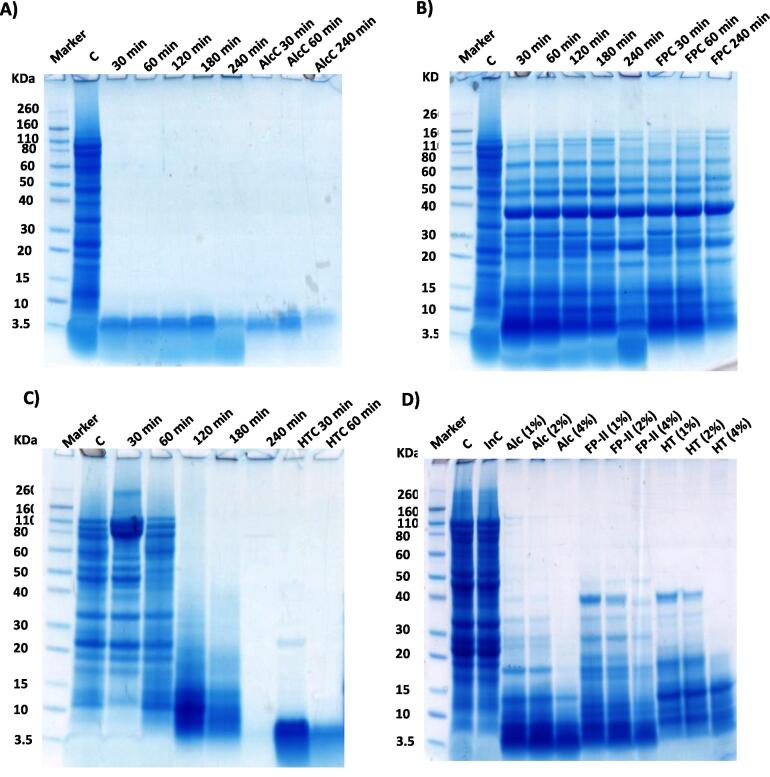
1D SDS-PAGE of protein hydrolysis profiles of hoki roe homogenate. (A) Alcalase, (B) FP-II, and (C) HT, all with the same amount of proteolytic activity (30000 U) and (D) effect of protease concentration (1 %, 2 %, 4 %) on protein hydrolysis of hoki roe homogenate after 3 h incubation under conditions stated in Materials and Methods. Abbreviations: C, hoki roe substrate without protease preparation; AlcC = Alcalase control; FPC = FP-II protease control; HTC = HT protease control.

The protein hydrolysis profiles of hoki roe homogenate samples hydrolysed for 3 h by Alcalase, HT and FP-II protease preparations were analyzed by 1D-SDS-PAGE to compare the time course degree of hydrolysis, as shown in Fig. 3A, 3B and 3C, with the last three lanes of each gel representing protease-only control. Several protein bands are evident on SDS-PAGE in FP-II-only control lanes (Fig. 3B), indicating that at the level of FP-II used in the hydrolysis corresponding to 30000U, there is a considerable contribution of protein from the FP-II, compared to that of the HT and Alcalase protease preparations. All three protease preparations used in the present study hydrolysed roe proteins in a non-selective manner, suggesting no apparent specificity for any of the roe proteins. The results indicate that Alcalase was more effective in hydrolysis of hoki roe homogenate than HT and FP-II protease, in agreement with the degree of hydrolysis results obtained, that is based on measuring generation of amino groups as a result of hydrolysis of peptide bonds (Fig. S1). Alcalase was found to hydrolyse *Cirrhinus cirrhosus* egg protein more effectively and produce more smaller peptides compared to papain, as indicated by SDS-PAGE (Chalamaiah, Rao, Rao, & Jyothirmayi, 2010), which is similar to the findings of the present study with hoki roe.

Alcalase has also been reported to efficiently produce small peptides on hydrolysis of stone fish protein, based on SDS-PAGE analysis (Bordbar et al., 2018). The absence of protein bands on SDS-PAGE for the Alcalase hydrolysis after 30 min (Fig. 3A), suggests that extensive proteolysis had occurred, resulting in the disappearance of polypeptide bands of more than the 3.5 kDa resolving limit of the PAGE. A similar hydrolysis pattern with Alcalase on SDS-PAGE has also been reported by Rasli and Sarbon (2019), with hydrolysis of shortfin scad skin to form gelatine.

From the SDS-PAGE analysis (Fig. 3), the HT protease preparation was found to be more hydrolytically active on hoki roe homogenate compared to FP-II (Fig. 3B and 3C, respectively). This result correlates with the OPA assay degree of hydrolysis results (Fig. S1C). This result is also consistent with the findings of Ryder et al. (2015), who reported a higher connective tissue hydrolysis capability of HT compared to FP-II. Ryder et al. (2016) reported that meat myofibrillar protein was hydrolysed by the HT protease preparation more effectively than by FP-II, consistent with the present study.

Overall, the SDS-PAGE analysis of the protein hydrolysis profiles suggests that 3 h proteolysis of hoki roe protein using Alcalase and HT would be sufficient to achieve a substantial degree of hydrolysis. The hoki roe homogenate substrate (20 g wet weight equivalent) was further hydrolysed with Alcalase, FP-II and HT for 3 h at pH 7.0 and 45 °C with three different protease:substrate proportions of 1, 2 and 4 %, Alcalase (200, 400, and 800 µL); HT (200 mg, 400 mg, or 800 mg) and FP-II (200 mg, 400 mg, or 800 mg), which are the most common proportions used and reported in the literature (Mahmoud et al., 2008, Normah et al., 2005, Shu et al., 2016). SDS-PAGE showed that after 3 h a higher degree of hydrolysis was achieved with an increase in protease concentration. This result is consistent with the findings of Normah (2005), where they found more protein hydrolysis occurred with an increase in the amount of protease used.

### Lipidomic profiling of lipid extracted from hoki roe homogenate hydrolysates

#### Lipid extract yield from hoki roe homogenate hydrolysates

The results indicate that a significant (*p* < 0.05) increase in the total lipid yield was achieved with all of the hoki roe homogenate hydrolysates evaluated, compared to the controls. The lipid yield from the controls is consistent with previous findings (Ahmmed, Bunga et al., 2020), where a lipid extract yield of ∼14 g from 100 g wet tissue was obtained from hoki roe homogenate. The lipid extract yield obtained from the ‘incubation controls’ that were incubated at 45 °C without protease, did not vary significantly (*p* > 0.05) from the lipid extract yield obtained from the ‘non-incubated controls’ (Fig. S2). This result indicates that the significant effects found with the treatments with protease are due to the proteolytic action of the protease preparations, and the incubation temperature is not directly influencing the lipid yield. Hydrolysis of protein in lipid-containing natural product materials may allow better accessibility of organic solvents, enabling higher extracted lipid yield (Dumay et al., 2006, Kechaou et al., 2009). Such proteolytic hydrolysis may generate some peptides that are lipophilic which may partition into the extracted lipid fraction (Kristinsson & Rasco, 2000).

A higher lipid extract yield was obtained from hoki roe homogenate hydrolysed with 4 % Alcalase, compared to 1 % and 2 % Alcalase (Fig. S2), indicating that a high degree of hydrolysis enhances lipid extraction. However, no significant difference (*p* < 0.05) in the yield of the extracted lipid was observed when hoki roe homogenate was hydrolysed using 2 % or 4 % HT or FP-II. There have been no reports of Alcalase hydrolysis of hoki roe homogenate prior to lipid extraction. Hydrolysis of rainbow trout roe using Alcalase has been reported to generate a higher total lipid yield (38.5 %), compared to that achieved after either Protamex hydrolysis (29.6 %) or Neutrase hydrolysis (18.3 %) (Mahmoud et al., 2008). Another study also obtained a higher total lipid extract yield (19.6 %) from salmon head with Alcalase, compared to Neutrase (14.4 %) and Protamex (14.6 %) (Gbogouri et al., 2006). A higher lipid extract yield was also obtained after Alcalase assisted hydrolysis of sardine viscera compared to use of only a solvent extraction method (Dumay et al., 2006). All of these results suggest that Alcalase has a high capability to hydrolyse protein in fish and fish co-products, resulting in enhanced lipid extract yield that was found to be better than that achieved by hydrolysis with HT and FP-II protease preparations.

#### TBARS and peroxide value (PV)

TBARS are co-products of lipid peroxidation, which can be detected using thiobarbituric acid as a reagent, and the assay is known as the TBARS assay (Xia et al., 2009). This analysis was carried out to investigate an aspect of the lipid oxidation status of the extracted lipid. Fig. S3A and S3B represent the TBARS and PV of the extracted total lipid obtained after proteolytic hydrolysis of hoki roe homogenates using the three different protease preparations (Alcalase, FP-II and HT). The results indicate that there is significant (*p* < 0.05) variation in TBARS and PV among the hydrolysis treatments. The incubated control samples were found to have higher TBARS than the samples hydrolysed with Alcalase. This result indicates that the Alcalase hydrolysed samples may contain some peptides that act as antioxidants and hence protected the lipid from oxidation. It has been reported that peptides of 500–1500 Da molecular weight are often more effective as antioxidants than peptides of more than 1500 Da (Li et al., 2008). Alcalase hydrolysis may generate peptides with enhanced antioxidant bioactivity (Tacias-Pascacio et al, 2020), which may explain why lipid extracted from roe after Alcalase hydrolysis exhibited a lower extent of oxidation compared to that obtained after HT or FP-II hydrolysis.

Protein hydrolysates produced from common carp roe by Alcalase were found to have a higher antioxidant activity compared to hydrolysates prepared using either trypsin or pepsin (Chalamaiah, Jyothirmayi, Diwan, & Dinesh Kumar, 2015). Addition of Alcalase generated gelatine hydrolysates into an unwashed mincemeat system reduced the TBARS significantly (*p* < 0.05) compared to control (Nikoo, Benjakul, & Xu, 2015). The antioxidant activity of peptides might be attributed to the metal-chelating ability, scavenging of oxygen-containing compounds, or reduction of free-radicals formed during lipid peroxidation (Kim & Wijesekara, 2010). Chicken egg derived peptides are reported to exhibit antioxidant activity against lipid peroxidation and scavenging of free radicals (Duan et al., 2014). This might explain why the hoki roe homogenate incubated control samples had higher TBARS values, compared to the Alcalase treated samples (Fig. S3A). Even though peptides were generated (as shown in Fig. 3) with HT and FP-II hydrolysis of hoki roe homogenate samples, the hydrolysate did not appear to protect the lipids from oxidation. This suggests that peptides generated by HT and FP-II might have different structure and functionality from the peptides generated by Alcalase, and peptides may differ in their antioxidant capability. The antioxidant characteristics of the peptide hydrolysates could be investigated in future research.

The peroxide value of the Alcalase hydrolysed hoki roe homogenate samples was found to be lower than that of the FP-II and HT protease hydrolysed sample. As mentioned earlier, Alcalase might produce peptides with antioxidant properties, which inhibit lipid peroxidation, and hence result in a lower PV value compared to that of HT and FP-II hydrolysates (Fig. S3B). This finding is corelated with the TBARS result, which indicates a higher lipid stability is achieved following Alcalase hydrolysis of hoki roe homogenate samples, compared to FP-II and HT (Fig. S3). Overall, both TBARS (<5 mg MDA/kg lipid) and PV value (<5 meq peroxide/kg) of lipid obtained from different treatments were within the acceptable limit and hence the extracted lipid can be considered to be of good quality, based on that previously reported (Guillen-Sanchez et al., 2020, Öz, 2018).

#### FAME analysis

The results of the FAME analysis presented in Table 2 indicate that there are significant differences (*p* < 0.05) among most of the fatty acids between protease-treated hoki roe homogenate samples and controls. However, γ-linolenic acid (C18:3), eicosenoic acid (C20:1), heneicosanoic acid (C21:0), docosatetraenoic acid (C22:4), tricosanoic acid (C23:0) and the n-3/n6 ratio were not different across the samples (Table 2). The present study also found that the non-incubated control (without incubation and protease added) was not different from the incubated control (incubated at 45 °C and no protease) (Table 2). As there was no apparent effect on the amount of unsaturated fatty acids, as a result of protein hydrolysis, the results suggest that incubation at 45 °C did not have any significant effect (*p* < 0.05) on the observed differences in fatty acids in treatment groups. Control groups were found to contain similar total saturated fatty acids, monounsaturated fatty acids (MUFA), polyunsaturated fatty acids (PUFA) and total n-3, compared to the HT protease treated samples (Table 2). However, the amount of the total SFA, MUFA, PUFA, total n-3 and total n-6 were decreased substantially with an increase of Alcalase concentration. Palmitic acid (C16:0) content was found to be the dominant saturated fatty acid in all of the samples. This fatty acid was found to vary significantly (*p* < 0.05) depending on the specific protease, protease concentration and their interaction. Alcalase treated hoki roe homogenate samples were found to contain less palmitic acid compared to the controls. This is in agreement with Mahmoud et al. (2008), who found less palmitic acid with enzyme assisted extraction (13.7 %), compared to solvent extraction (16.1 %). In the present study, the palmitic acid content was decreased with the increasing amount of Alcalase used in the hydrolysis step. The higher amount of protease preparations used might produce a higher amount of lipophilic polypeptides, which contributed the total weight of the lipid, resulting in an apparent lower palmitic acid content with the increase of protease percentage. Oleic acid (C18:1) was found to be the most dominant monounsaturated fatty acid (MUFA) in all hoki roe lipid extract samples analyzed in the present study, which is consistent with findings presented previously (Ahmmed, Bunga et al., 2020). The oleic acid content in protease treated samples also varied significantly (*p* < 0.05) from the controls. The results indicate a significant decrease (*p* < 0.05) in oleic acid content with an increase in the amount of Alcalase.

**Table 2:** Fatty acid composition (mg/100 mg lipid) in total lipid extracted from controls and protease hydrolysed hoki roe homogenate samples.

Protease Level	Control	InC^1^	Alcalase			FP-II protease			HT protease			SEM	*p* value		
			**1 %**	**2 %**	**4 %**	**1 %**	**2 %**	**4 %**	**1 %**	**2 %**	**4 %**		E	%EC	E * EC
C14:0	0.95^A^	0.91^A^	0.78^BC^	0.79^BC^	0.54^C^	0.77^AB^	0.78^AB^	0.78^AB^	0.87^A^	0.99^A^	0.84^A^	0.05	0.001	0.034	0.011
C14:1	0.08^A^	0.07^A^	0.06^AB^	0.06^AB^	0.04^B^	0.06^AB^	0.06^AB^	0.06^AB^	0.07^AB^	0.08^A^	0.05^AB^	0.01	0.016	0.053	0.197
C16:0	5.57^A^	5.43^A^	4.66^BC^	4.69^BC^	3.31^C^	4.78^AB^	4.85^AB^	4.72^ABC^	5.05^A^	5.86^A^	5.91^A^	0.28	0.001	0.006	0.005
C16:1 n7	1.71^A^	1.68^A^	1.43^B^	1.44^B^	0.99^C^	1.40^B^	1.41^B^	1.40^B^	1.56^AB^	1.79^A^	1.62^AB^	0.08	0.001	0.001	0.001
C17:0	0.09^AB^	0.09^AB^	0.07^BC^	0.08^BC^	0.06^C^	0.08^ABC^	0.08^ABC^	0.08^ABC^	0.08^AB^	0.09^AB^	0.10^A^	0.01	0.001	0.046	0.029
C17:1n-8	0.23^A^	0.23^A^	0.18^BC^	0.18^BC^	0.12^D^	0.18^BC^	0.18^BC^	0.18^BC^	0.20^ABC^	0.23^A^	0.22^A^	0.01	0.001	0.044	0.002
C18:0	0.76^ABC^	0.74^ABC^	0.61^BC^	0.63^BC^	0.47^C^	0.68^ABC^	0.70^ABC^	0.66^ABC^	0.64^ABC^	0.79^AB^	0.93^A^	0.06	0.001	0.343	0.076
C18:1 n9	8.71^AB^	9.71^A^	7.08^ABC^	7.19^ABC^	4.87^C^	7.02^BC^	7.07^BC^	7.00^BC^	7.77^AB^	8.94^AB^	9.49^AB^	0.50	0.001	0.016	0.009
C18:1 n7	1.24^A^	1.12^AB^	0.92^BC^	0.93^ABC^	0.63^C^	0.91^BC^	0.92^BC^	0.90^BC^	0.99A^B^	1.15^AB^	1.22^AB^	0.06	0.001	0.034	0.011
C18:2 n6 trans	0.09^A^	0.08^AB^	0.07^ABC^	0.06^BC^	0.04^C^	0.06^BC^	0.07^ABC^	0.07^ABC^	0.07^ABC^	0.08^AB^	0.08^AB^	0.01	0.001	0.022	0.016
C18:2 n6 cis	0.49^A^	0.44^ABC^	0.38^BC^	0.38^BC^	0.26^D^	0.37^BCD^	0.37^BC^	0.35^CD^	0.39^ABC^	0.46^ABC^	0.45A^BC^	0.02	0.001	0.004	0.002
C18:3 n6	0.05	0.05	0.03	0.04	0.03	0.04	0.03	0.04	0.04	0.04	0.04	0.03	0.061	0.036	0.060
C18:3n-3	0.21^A^	0.20^A^	0.17^BCD^	0.17^BCD^	0.12^D^	0.17^BCD^	0.17^BCD^	0.16^CD^	0.18^ABC^	0.21^A^	0.20^A^	0.01	0.001	0.004	0.009
C20:1n-11	0.24	0.23	0.20	0.20	0.14	0.19	0.19	0.17	0.21	0.24	0.21	0.05	0.063	0.347	0.157
C20:2 n6	0.11	0.10	0.08	0.08	0.05	0.08	0.08	0.08	0.09	0.10	0.10	0.11	0.074	0.334	0.241
C21:0	0.09	0.08	0.07	0.07	0.05	0.07	0.07	0.07	0.08	0.09	0.10	0.06	0.084	0.591	0.028
C20:3n-3	0.30^A^	0.29^AB^	0.25^ABC^	0.25^ABC^	0.18^C^	0.25^ABC^	0.26^ABC^	0.21^BC^	0.24^ABC^	0.29^AB^	0.31^A^	0.02	0.001	0.127	0.024
C20:4 n6	0.09	0.09	0.08	0.08	0.05	0.07	0.07	0.07	0.08	0.09	0.10	0.06	0.101	0.210	0.004
C22:0	0.55^A^	0.48^ABC^	0.41^BCD^	0.41^BCD^	0.29^E^	0.39^CDE^	0.40^CDE^	0.36^DE^	0.43^BCD^	0.50^ABC^	0.49^ABC^	0.02	0.001	0.004	0.002
C22:1 n9	0.15^AB^	0.13^AB^	0.11^AB^	0.12^AB^	0.07^B^	0.11^AB^	0.11^AB^	0.11^AB^	0.13^AB^	0.15^AB^	0.19^A^	0.02	0.017	0.566	0.480
C20:5n-3 [EPA]	2.63^A^	2.38^AB^	2.04^ABC^	2.02^ABC^	1.46^C^	1.97^ABC^	2.05^ABC^	1.75^BC^	2.05^ABC^	2.40^AB^	2.35^AB^	0.13	0.001	0.032	0.058
C23:0	0.09	0.08	0.07	0.07	0.05	0.07	0.07	0.06	0.08	0.09	0.09	0.13	0.101	0.086	0.001
C24:1n-9	0.05^B^	0.05^B^	0.04^B^	0.04^B^	ND	0.04^B^	0.04^B^	0.04^B^	0.04^B^	0.05B	0.09^A^	0.01	0.150	0.573	0.210
C22:5n-3 [DPA]	0.60^A^	0.52^ABC^	0.44^BCDE^	0.45^BCDE^	0.31^E^	0.43^CDE^	0.44^BCDE^	0.39^DE^	0.47^ABCD^	0.54^ABC^	0.58^AB^	0.03	0.001	0.027	0.003
C22:6n-3 [DHA]	7.01^A^	6.17^AB^	5.45^ABCD^	5.22^ABCD^	3.85^D^	5.10^BCD^	5.29^ABCD^	4.31^CD^	5.74^ABC^	6.54^AB^	7.01^A^	0.33	0.001	0.042	0.004
Unknown	2.83^A^	2.24^AB^	1.89^ABCD^	1.74^BCD^	1.30^D^	1.78^BCD^	1.86^ABCD^	1.75^BCD^	2.26^AB^	1.97^ABCD^	2.14^AB^	0.19	0.001	0.057	0.001
Total	36.8^A^	32.1^ABC^	27.6^BC^	27.4^BCD^	19.3^D^	27.1^BCD^	27.6^BC^	25.8^CD^	29.8^ABC^	33.8^ABC^	34.9^AB^	1.58	0.001	0.009	0.002
SFA	8.04^A^	7.81^A^	6.68^AB^	6.74^AB^	4.76^B^	6.84^AB^	6.94^AB^	6.73A^B^	7.22^A^	8.40^A^	8.45^A^	0.42	0.001	0.047	0.001
MUFA	12.3^A^	11.7^A^	10.1^AB^	10.2^AB^	6.86^C^	9.89^B^	9.98^B^	9.87^B^	11.0^AB^	12.7^A^	13.1^A^	0.71	0.001	0.038	0.011
PUFA	10.7^A^	10.3^A^	8.98^AB^	8.75A^B^	6.35^C^	8.54^AB^	8.83^AB^	7.43^B^	9.36^AB^	10.6^A^	11.2^A^	0.56	0.001	0.047	0.035
Total n-3	9.91^AB^	9.61^AB^	8.38^ABC^	8.15^ABC^	5.95^D^	7.95^BC^	8.24^ABC^	6.86^CD^	8.72^ABC^	10.0^A^	10.5^A^	0.52	0.001	0.027	0.028
Total n-6	0.78^A^	0.75^A^	0.63^B^	0.64^B^	0.43^C^	0.62^B^	0.63^B^	0.60^B^	0.68^AB^	0.78^A^	0.77^A^	0.04	0.001	0.013	0.017
n6/n-3	0.08	0.08	0.08	0.08	0.07	0.08	0.08	0.09	0.08	0.08	0.07	0.07	0.074	0.514	0.514

Abbreviations: Inc, incubated control (incubated hoki roe at 45 °C without protease preparation); E = protease; C = Protease concentration; ND = not detected; EPA = eicosapentaenoic acid; DPA = docosapentaenoic acid; DHA = docosahexaenoic acid; SFA = total saturated fatty acids; MUFA = total monounsaturated fatty acids; PUFA = total polyunsaturated fatty acids; SEM = Standard error mean; E = effect of protease; EC = effect of protease; E*EC = interaction effect. Each value represents the mean of triplicate samples. Different superscript letters (A–E) in the same row indicate a significant difference (*p* < 0.05). The lipid was extracted from freeze dried hoki roe (20 g roe wet weight equivalent), which was hydrolysed with different amount of protease preparations (Alcalase: 200, 400, or 800 µL; HT: 200, 400, or 800 mg and FP-II: 200, 400, or 800 mg), corresponding to 1 %, 2 % and 4 %, respectively.

The level of long chain n-3 fatty acids in hoki roe homogenate samples varied significantly (*p* < 0.05) depending on the protease used, the amount used and their interaction. The HT treated hoki roe homogenate sample was found to contain a higher amount of PUFA with bioactive n-3 fatty acids including EPA, DPA and DHA compared to Alcalase and FP-II (Table 2). The lower PUFA including EPA, DPA and DHA content in Alcalase treated samples compared to control is consistent with Mahmoud et al. (2008), who found a lower fatty acid content (EPA = 11.5 %; DPA = 4.8 %, DHA = 24.0 % and total n-3 = 40.3 %) in rainbow trout roe lipid extracted with solvent, compared to enzyme-assisted extraction by Alcalase (EPA = 11.3 %; DPA = 4.4 %;DHA = 19.0 % and total n-3 = 34.7 %). The results also indicate a decreasing trend of fatty acid content with a higher amount of Alcalase, which appears to be not related to lipid oxidation as the TBARS and PV values for the lipid extract obtained after Alcalase hydrolysis of hoki roe homogenate was lower compared to other treatments (Fig. S3). A higher degree of hydrolysis of hoki roe protein was obtained with Alcalase, as a result of generating more peptides compared to that achieved by FP-II and HT protease hydrolysis (Fig. S1). The highest concentration of Alcalase (4 %) used was also found to exhibit higher protein degradation capability followed by 2 % and 1 % Alcalase, as shown previously by SDS-PAGE analysis (Fig. 3D), It may be that the lipid extracted from Alcalase hydrolysed roe homogenate may contain lipid soluble polypeptide, as reported previously by Hidalgo et al. (2001), which would contribute to the total weight of the extracted lipid fraction. This might explain why fatty acid proportion of the lipid extract is appearing to decrease with the increase of protease concentration used for hydrolysis of the roe. Common carp fish roe protein hydrolysates were found to exhibit immunomodulatory effects in a female mouse model and stimulated both the humoral and cell-mediated immune response without any allergenicity (Chalamaiah, Hemalatha et al., 2015), indicating that the combination of the lipid with (bioactive) peptides could be more beneficial compared to the lipid without peptides. This would be worth further investigation in the future.

#### Determination of phospholipid composition by NMR

A comparative phospholipidomic profile is presented in Table 3, which shows that there is a significant variation (*p* < 0.05) in phospholipid content among the hoki roe homogenate treated samples. Phosphatidylcholine (PC) was the dominant phospholipid class in all samples analyzed in the present study, in agreement with the findings reported previously (Ahmmed, Bunga et al., 2020), where PC was found to be the dominant phospholipid class in hoki roe. The incubated control was found to contain a lower PC content compared to control samples. Hoki roe might contain some endogenous phospholipases (Ahmmed, Bunga et al., 2020), which could be active at the incubation temperature and hydrolyse the PC, This could be the reason for there being a lower PC content in incubated roe homogenate samples, compared to the non-incubated control (Table 3).

**Table 3:** Phospholipid composition (μmol/g wet tissue) in total lipid extracted from control and protease hydrolysed hoki roe homogenate samples.

PL	Control	Inc	Alcalase			FP-II protease			HT protease			SEM	*p* value		
			**1 %**	**2 %**	**4 %**	**1 %**	**2 %**	**4 %**	**1 %**	**2 %**	**4 %**		**E**	**%EC**	E* EC
PA	0.58^A^	0.61 ^A^	0.44^BC^	0.55^AB^	0.34^CD^	0.26^D^	0.33^CD^	ND	0.64^A^	0.46^BC^	0.44^BC^	0.05	0.001	0.001	0.001
LDPG	0.71^BC^	0.64^B^	0.38^BC^	0.30^BC^	0.08^BC^	0.21^BC^	0.08^C^	2.05^A^	0.47^BC^	0.39^BC^	0.41^BC^	0.15	0.004	0.001	0.001
CL	0.28	ND	ND	ND	ND	ND	ND	ND	ND	ND	ND	ND	NS	NS	NS
LPE	0.18^BC^	0.21^AB^	0.18^BC^	0.23^AB^	ND	ND	ND	ND	0.33^A^	ND	0.13^C^	0.03	0.001	0.002	0.001
LPS	5.34^A^	5.42^A^	2.87^CDE^	2.87^DE^	2.31^E^	2.47^C^	2.31^E^	ND	4.03^BCD^	3.42^CDE^	2.95^DE^	0.25	0.001	0.001	0.001
SM	1.24	0.47	ND	ND	ND	ND	ND	ND	ND	ND	ND	0.14	NS	NS	NS
PE	1.04	0.70	ND	ND	ND	ND	ND	ND	ND	ND	ND	0.13	NS	NS	NS
LPC	2.50^C^	1.38^C^	1.60^DE^	1.11 ^DE^	1.26^DE^	4.76^B^	5.90^B^	7.52^A^	2.21^CDE^	2.03^CDE^	1.88^CDE^	0.23	0.001	0.001	0.001
PS	0.76^B^	0.56^B^	0.65^B^	0.24^B^	0.46^B^	2.74^A^	3.74^A^	2.76^A^	0.55^B^	0.38^B^	0.58^B^	0.19	0.001	0.112	0.020
PI	1.08^A^	0.77A	0.84^AB^	0.83^AB^	0.86^AB^	0.79^ABC^	0.78^ABC^	ND	0.91^AB^	0.24^C^	0.79^ABC^	0.11	0.001	0.001	0.001
PC	26.40^A^	22.07^CD^	24.17^BC^	16.4^F^	17.05^F^	18.56^F^	18.16^F^	8.80^G^	28.35^A^	23.04^CDE^	23.50^CD^	0.44	0.001	0.002	0.001
Total	40.12^A^	32.82^AB^	31.59^AB^	22.6^C^	22.6^C^	29.8^AB^	31.30^AB^	21.13^C^	37.49^A^	29.97^AB^	30.69^AB^	0.43	0.001	0.001	0.002

Abbreviations: PL + phospholipid; PA = phosphatidic acid; LDPG = lyso-diphosphatidylglycerol; CL = cardiolipin; LPE = lyso-phosphatidylethanolamine; LPS = lyso-phosphatidylserine; SM = sphingomyelin; PE = phosphatidylethanolamine; LPC = lyso-phosphatidylcholine; PS = phosphatidylserine; PI = phosphatidylinositol; PC = phosphatidylcholine; ND = not detected; and NS = not significant; Control = sample without incubation and protease pre-treatment; Inc = sample incubated at 45 °C without protease preparations; SEM = Standard error mean; E = effect of protease; EC = effect of protease; E*EC = interaction effect. Each value represents the mean of triplicate samples. Different superscript letters (A–E) in the same row indicate a significant difference (*p* < 0.05). The lipid was extracted from free dried hoki roe (20 g roe wet weight equivalent), which was hydrolysed with different amount of protease preparations (Alcalase: 200, 400, or 800 µL; HT: 200, 400, or 800 mg and FP-II: 200, 400, or 800 mg), corresponding to 1 %, 2 % and 4 %, respectively.

The result found higher phospholipid content in HT pre-treated hoki roe compared to Alcalase and FP-II pre-treated samples. Again, total phospholipid content including LPS, LPC and PC were higher with 1 % protease pre-treated samples compared to 4 % of the same protease preparations. The additional decreasing trend of phospholipid content with the increasing level of the protease preparations (Alcalase, FP-II and HT), might be related to some peptide material partitioning with the lipid fractions. In the present study, a hexane/ethanol based solvent extraction method (ETHEX) was used, which does not create any aqueous layer like chloroform/methanol/water solvent in the FOLCH or Bligh & Dyer method, resulting in water soluble or lipid soluble peptides not being partitioned away from the lipid fraction in the ETHEX method. Analysis aimed at detecting peptides present in the lipid extract would be an interesting area of further investigation. The higher levels of protease correlated with a higher degree of hydrolysis (Fig. 2), resulting in more low molecular weight peptides being present in the hydrolysates (Fig. 3). As mentioned earlier, due to the particular lipid extraction method used, it may be that lipophilic peptides may have partitioned into the lipid fractions. This could have contributed to the total weight of the samples used in the NMR analyses, contributing to the decreasing trend of phospholipid content with the increasing amount of protease used.

## Conclusion

The present study characterized two bacterial protease preparations (Alcalase, HT) and one fungal protease preparation (FP-II) and investigated their hydrolytic ability and effect on the extraction of total lipid from hoki roe, and the lipid quality (fatty acid composition, phospholipid profile, PV, TBARS). The hydrolyses of hoki roe homogenate with the three proteases indicated differences in the rate of hydrolysis compared to casein, with Alcalase achieving the most extensive hydrolysis of the hoki roe protein. The yield of extracted lipid was found to increase following roe protein hydrolysis. Although Alcalase hydrolysis was found to result in a higher extracted lipid yield than HT and FPI-II, the fatty acid and phospholipid content recovered with HT hydrolysed samples was of better quality. Interestingly, the Alcalase hydrolysate appeared to afford antioxidant protection to the lipid, possibly due to the more extensive protein hydrolysis achieved that likely generated more antioxidant peptides, which could be investigated further in a future study. Overall, the present study indicates that extracted lipid yield can be enhanced as a result of roe protein hydrolysis with both Alcalase and HT, but food grade HT would be a more appropriate choice for obtaining lipid extracts enhanced in marine n-3 fatty acid and phospholipid content.

## Funding information

This work was supported by Sanford Limited, Auckland [Grant No: 18327].

## Declaration of Competing Interest

The authors declare the following financial interests/personal relationships which may be considered as potential competing interests: Alaa Bekhit reports a relationship with University of Otago that includes: employment.

## Data Availability

Data will be made available on request.
