# Supplementary material for: Use of fungal and bacterial protease preparations to enhance extraction of lipid from fish roe: Effect on lipidomic profile of extracted oil
Source: Food Chem X. 2022 Nov 8;16:100499. doi: 10.1016/j.fochx.2022.100499 (PMC9663326; doi:10.1016/j.fochx.2022.100499)
Supplement: Supplementary data 1 [file mmc1.docx]

Table S1: Preparation of protease stock solutions of different protease units

| Protease Units | Alcalase (μL/mL)^1^ | FP-II (mg/mL) | HT (mg/mL) |
| --- | --- | --- | --- |
| 1500 | **10** | 21.25 | 12.24 |
| 15000 | **100** | 212.5 | 122.4 |
| 30000 | **200** | 425 | 244.8 |

^1^Alcalase was available as a solution, whereas FP-II and HT were supplied as a powder


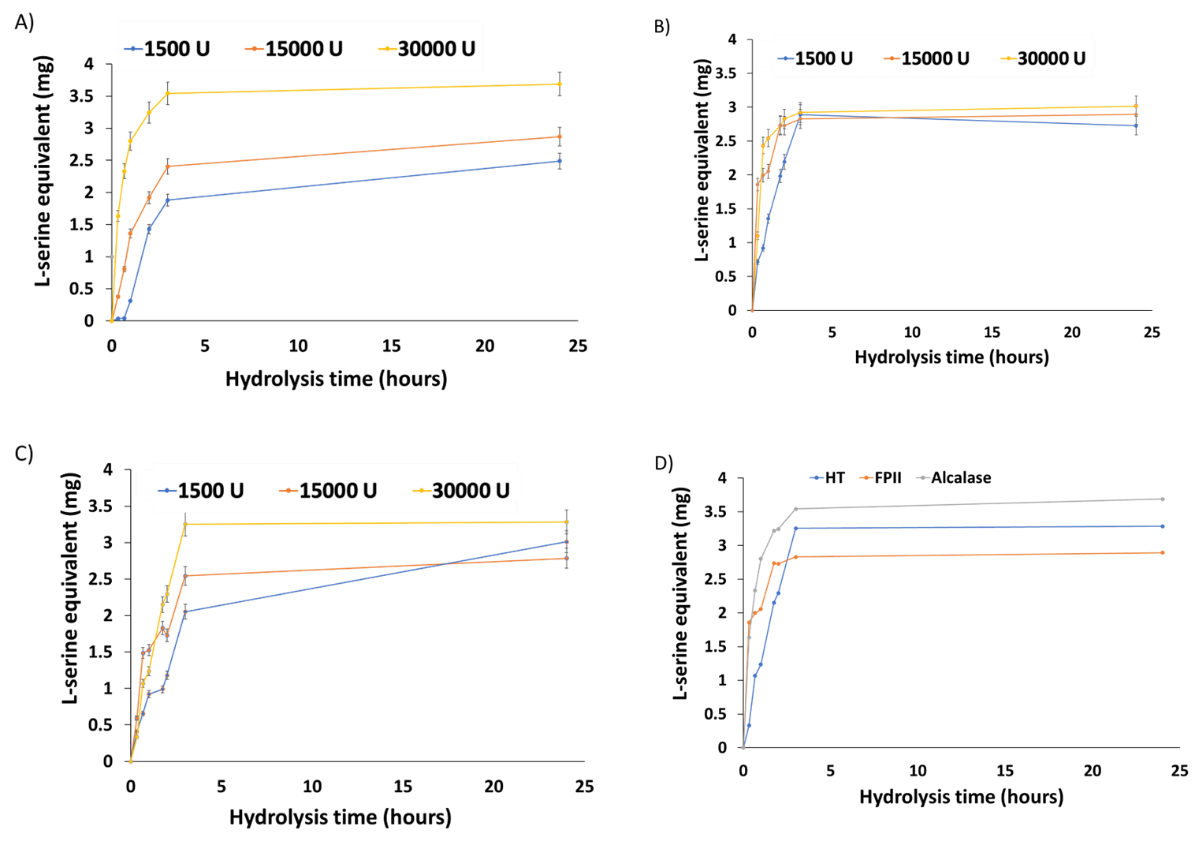


Figure S1. Degree of hydrolysis time course of hoki roe homogenate using three different amounts of each protease preparation (1500 U, 15000 U and 30000 U). (A) Alcalase, (B) FP-II, (C) HT, and (D) comparison of the L-serine equivalents released from hoki roe by the three protease hydrolyses under conditions stated in Materials and Methods. The L-serine equivalent released (mg) increased with increase in the amount of protease preparation, regardless of the enzyme. Maximum hydrolysis was obtained within 3 h for all cases. Alcalase was found to produce a higher L-serine equivalent released (mg) followed by HT and FP-II (D).

Figure S2. Total lipid extract yield (g/100g wet tissue) obtained from protease hydrolysed and un-treated hoki roe homogenate samples. Abbreviations: Incu-Con = sample incubated at 45°C without protease preparation; Alc = Alcalase; different letters (A-D) indicate significant difference (*p* < 0.05) among the treatments. The protease preparations (Alcalase: 200, 400, 800 µL; HT: 200, 400, 800 mg and FP-II: 200, 400, 800 mg) were used for 20 g wet hoki roe substrate. Different letters (A-C) on the bar indicate significant differences (*p* <0.05) in lipid content among the treatments.

Figure S3. TBARS (mg MDA/kg lipid) (A) and peroxide value (PV) (meq peroxide/kg) (B) lipid extracted from controls and samples hydrolysed with Alcalase, FP-II and HT protease preparations at specific different concentrations (1, 2 and 4%). Abbreviations: C1 substrate without incubation and protease pre-treatment; EnC1, sample incubated at 45°C without protease preparations; Alc = Alcalase; Different superscript letters indicate significant difference (*p* < 0.05) among the treatments. The lipid was extracted from freeze dried hoki roe (20 g roe wet weight equivalent), which was hydrolysed with different amount of protease preparations (Alcalase: 200, 400, or 800 µL; HT: 200, 400, or 800 mg and FP-II: 200, 400, or 800 mg). Different letters (A-C) on the bar indicates significant differences (*p* <0.05) among the treatments.
